# Supplementary material for: Efficacy and Microbiota Modulation Induced by LimpiAL 2.5%, a New Medical Device for the Inverse Psoriasis Treatment
Source: Int J Mol Sci. 2023 Mar 28;24(7):6339. doi: 10.3390/ijms24076339 (PMC10094358; doi:10.3390/ijms24076339)
Supplement: Supplementary file 1 [file ijms-24-06339-s001.zip › ijms-2280360-supplementary.pdf]

**SUPPLEMENTARY FIGURES AND TABLES.**

**Table S1.** LimpiAL 2.5% topical cream formula.

| Ingredients | Concentration (%) |
|---|---|
| water | 64.05 % |
| hydrated HAc-40 (85-86% water, 14-15% hyaluronic acid, i.e. HA, conjugated with <1% of bacterial cell wall fragment, i.e. c-40) | 17.85 % |
| | |
| caprylic/capric triglyceride | 3 % |
| *Chamomilla recutita* flower oil extract | 3 % |
| *Helichrysum italicum* glyceric extract | 3 % |
| Sepilife™ nude | 2.75 % |
| Fluidifeel™ easy | 2 % |
| ammonium lactate | 1 % |
| glycerin | 1 % |
| glyceryl caprylate | 0.80 % |
| butyrospermum parkii butter | 0.50 % |
| decylene glycol | 0.40 % |
| tocopheryl acetate | 0.30 % |
| sodium hydroxide | 0.25 % |
| zinc gluconate | 0.10 % |

**Table S2.** Statistical comparison between the sequencing outputs of NGS Illumina MiSeq. In the first column are reported the statistical significance values (p-values) obtained comparing the reads number of the indicated comparisons. In the second column are reported the statistical significance values (p-values) regarding the number of OTUs and calculated through the ANOVA test.

| Comparison | **Reads** *Kolmogorov-Smirnov test (p-value)* | **OTUs** *ANOVA (p-value)* |
|---|---|---|
| **T0** *vs* **CTRL** | 0.36 | 0.32 |
| **T1** *vs* **CTRL** | 0.74 | 0.34 |
| **T1** *vs* **T0** | 0.40 | 0.90 |

**Table S3**. Statistical comparison of the alpha diversity indexes between the indicated comparisons and taxonomic levels. The blue values indicate the comparison was performed through the Kolmogorov-Smirnov test (p ≤0.05). The black ones were calculated using the Whelch's t-test (p ≤0.05).

| | **OTUs number** | | | **Chao1** | | | **Shannon** | | |
|---|---|---|---|---|---|---|---|---|---|
| **Taxon** | T0 *vs* CTRL | T1 *vs* CTRL | T1 *vs* T0 | T0 *vs* CTRL | T1 *vs* CTRL | T1 *vs* T0 | T0 *vs* CTRL | T1 *vs* CTRL | T1 *vs* T0 |
| ***Phylum*** | 0.097 | 0.147 | 0.345 | 0.945 | 0.100 | 0.099 | 0.452 | 0.426 | 0.603 |
| ***Class*** | 0.107 | 0.330 | 0.512 | 0.646 | 0.816 | 0.400 | 0.358 | 0.361 | 0.250 |
| ***Order*** | 0.086 | 0.368 | 0.726 | 0.591 | 0.982 | 0.811 | 0.358 | 0.127 | 0.537 |
| ***Family*** | 0.132 | 0.346 | 0.863 | 0.137 | 0.063 | 0.759 | 0.358 | 0.127 | 0.250 |
| ***Genus*** | 0.081 | 0.279 | 0.795 | 0.054 | 0.352 | 0.889 | 0.591 | 0.444 | 0.933 |
| ***Species*** | 0.089 | 0.324 | 0.785 | 0.093 | 0.314 | 0.715 | 0.078 | 0.244 | 0.533 |

**Table S4.** Taxa differentially modulated in the indicated compared microbiotas (DESeq2, p-value and FDR ≤ 0.05)

| **Comparison** | **Modulated taxon** | **OTUs ID** | **logFC** | **p-value** | **FDR** | **logFC.std** |
|---|---|---|---|---|---|---|
| **T0 *vs* CTR** | Class | *Bacteroidetes;Cytophagia* | -3.0623 | 0.0002 | 0.0255 | -3.4926 |
| **T1 *vs* CTR** | Phylum | *Firmicutes* | 2.6131 | 0.0013 | 0.0465 | -1.2585 |
| | Class | *Firmicutes;Bacilli* | 2.6078 | 0.0004 | 0.0377 | 2.1609 |
| | Order | *Firmicutes;Bacilli;Bacillales* | 3.7885 | 4.34E-05 | 0.0054 | 2.6236 |
| | Order | *Tenericutes;Mollicutes;Mycoplasmatales* | 5.8157 | 9.03E-05 | 0.0075 | 4.8276 |
| | Order | *Actinobacteria;Actinobacteria;Actinomycetales* | 3.5652 | 0.0003 | 0.0208 | 3.2728 |

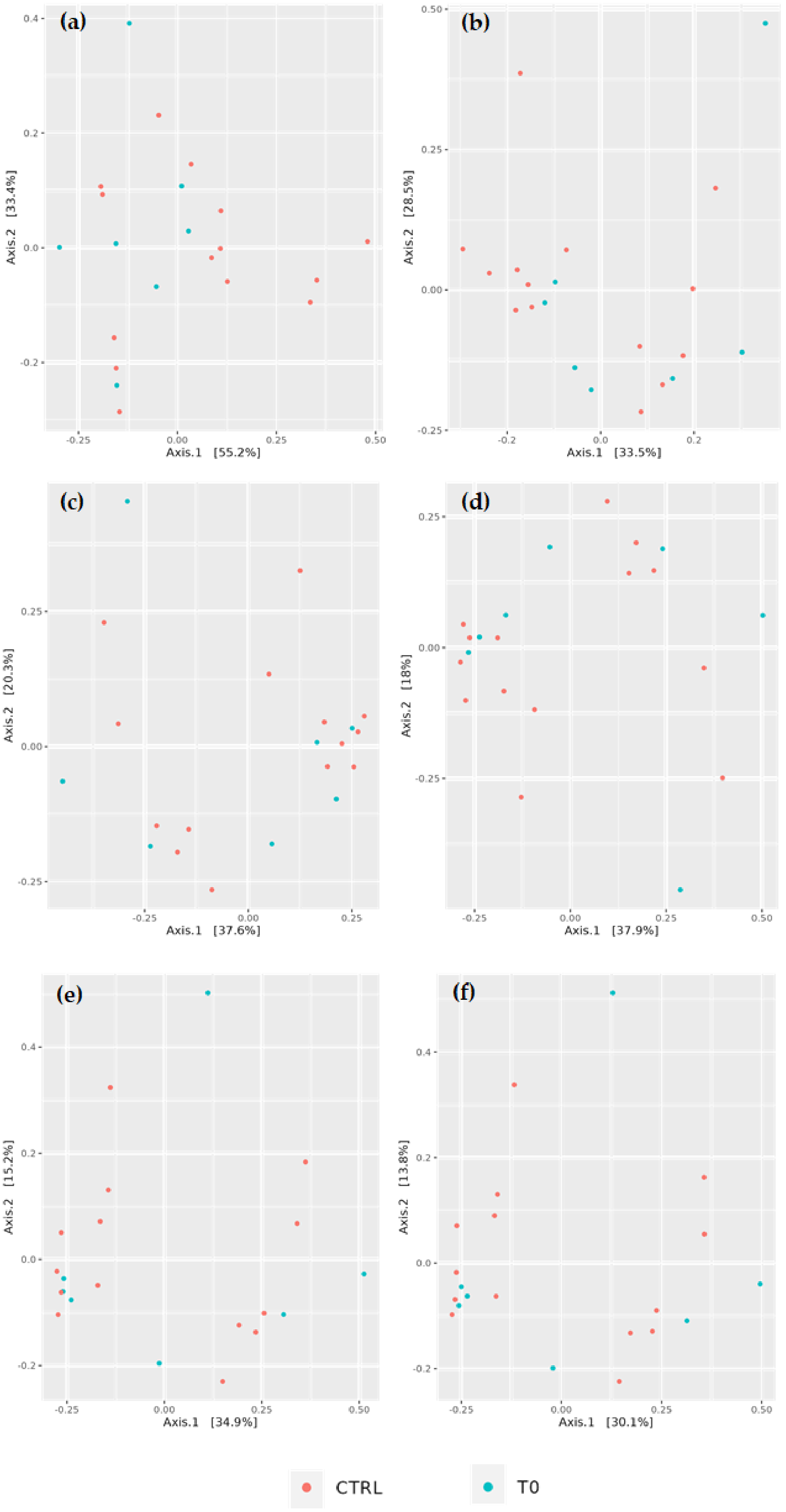


**Figure S1.** PCoA of Bray-Curtis distances between the T0 and CTRL samples at phylum (a), class (b), order (c), family (d), genus (e) and species (f) level.

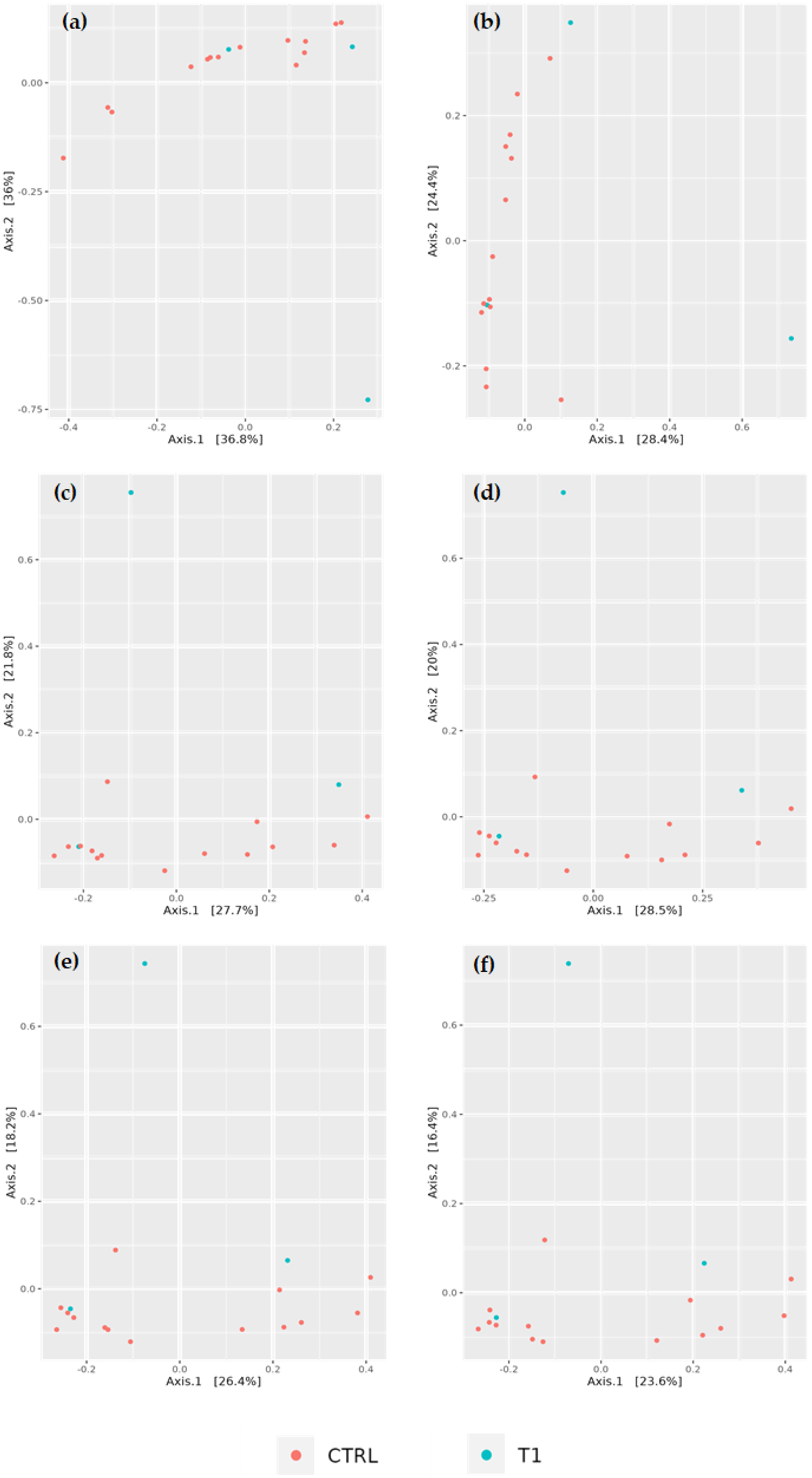


**Figure S2.** PCoA of Bray-Curtis distances between the T1 and CTRL samples at phylum (a), class (b), order (c), family (d), genus (e) and species (f) level.

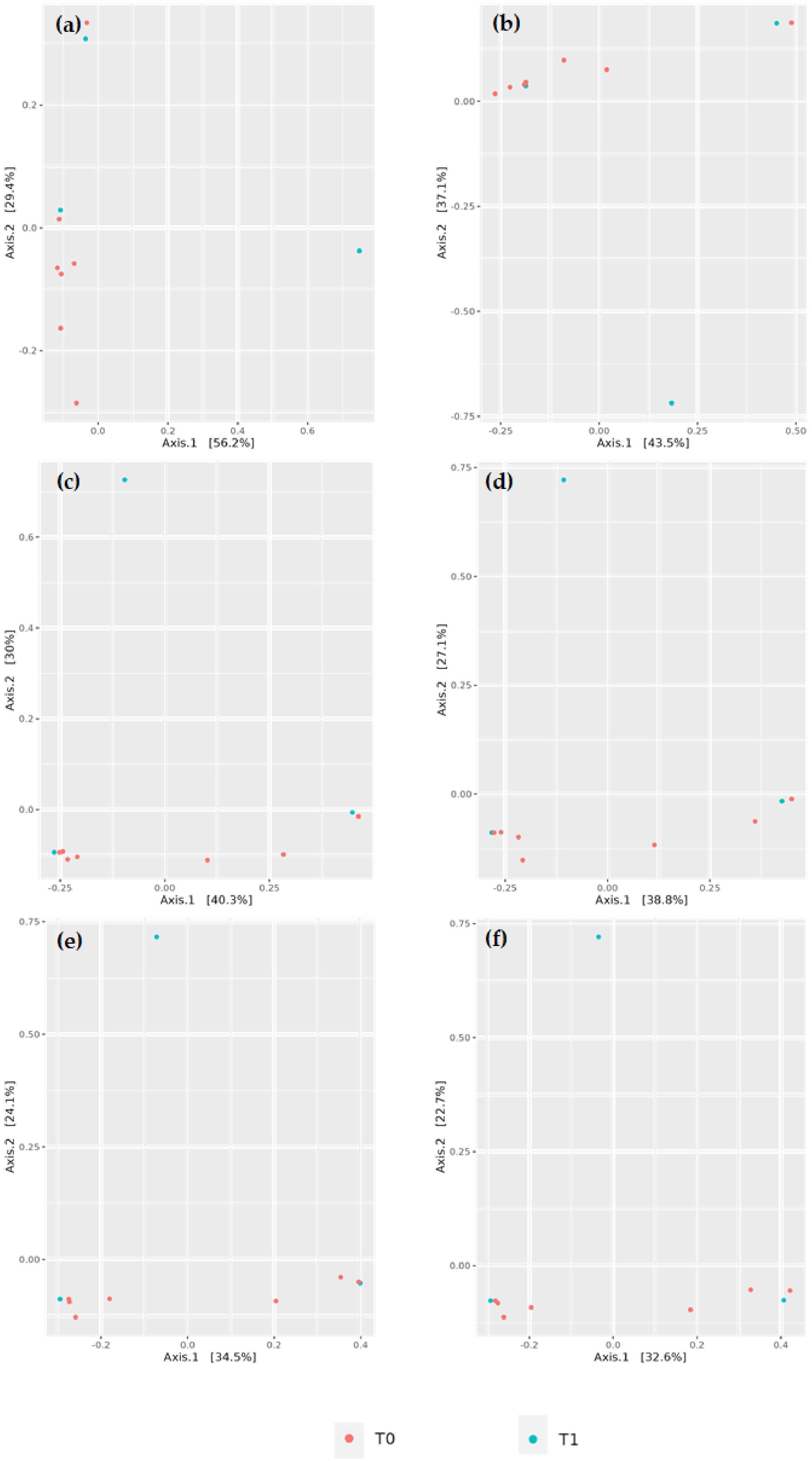


**Figure S3.** PCoA of Bray-Curtis distances between the T1 and T0 samples at phylum (a), class (b), order (c), family (d), genus (e) and species (f) level.

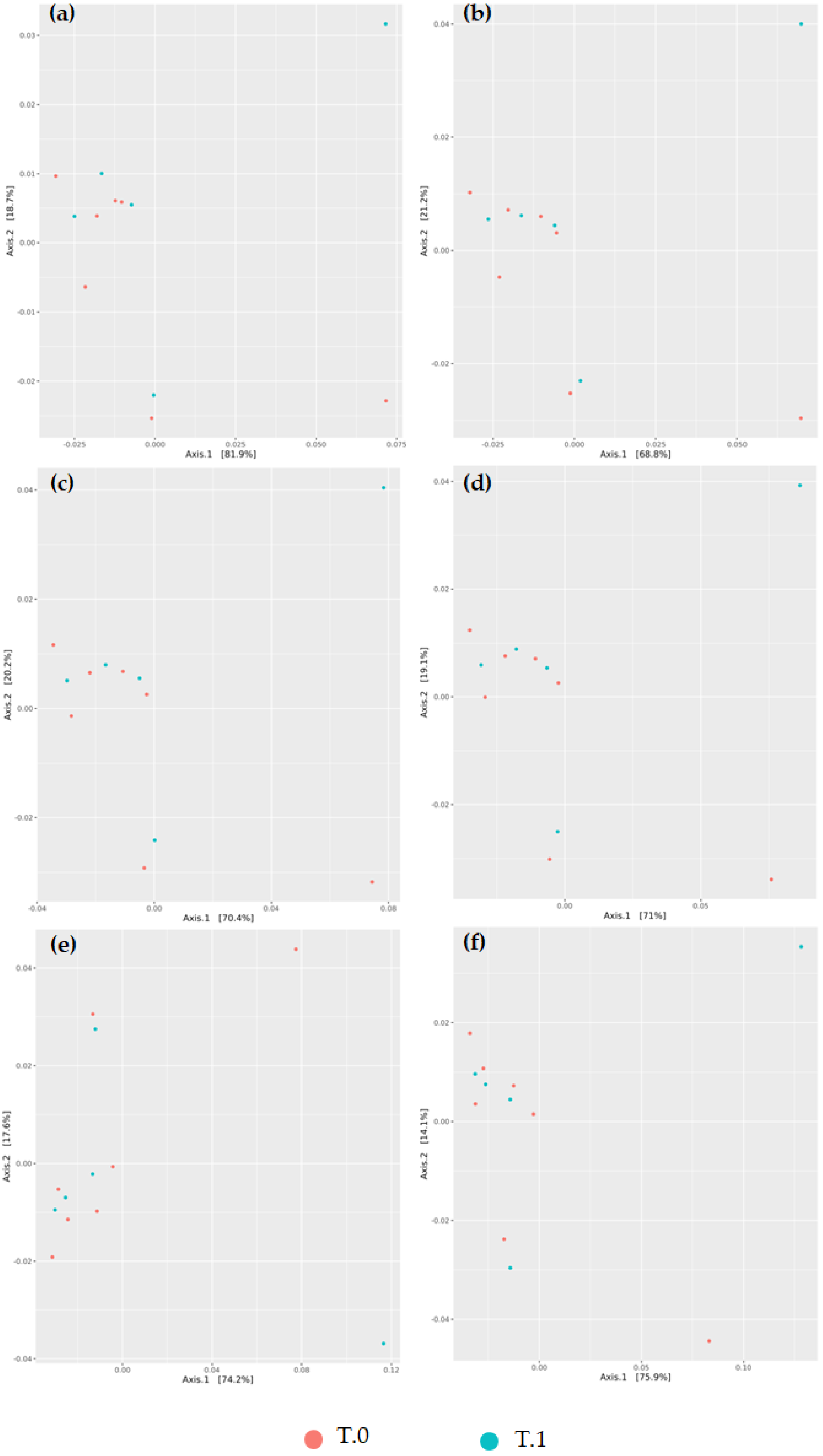


**Figure S4.** PCoA of Bray-Curtis distances between the T.1 and T.0 samples at phylum (a), class (b), order (c), family (d), genus (e) and species (f) level.
